# 17q Gain in Neuroblastoma: A Review of Clinical and Biological Implications

**DOI:** 10.3390/cancers16020338

**Published:** 2024-01-12

**Authors:** Vid Mlakar, Isabelle Dupanloup, Fanny Gonzales, Danai Papangelopoulou, Marc Ansari, Fabienne Gumy-Pause

**Affiliations:** 1 Cansearch Research Platform for Pediatric Oncology and Hematology, Faculty of Medicine, Department of Pediatrics, Gynecology and Obstetrics, University of Geneva, Rue Michel Servet 1, 1211 Geneva, Switzerland; isabelle.dupanloup@unige.ch (I.D.); fanny.gonzales@unige.ch (F.G.); danai.papangelopoulou@hcuge.ch (D.P.); marc.ansari@hcuge.ch (M.A.); fabienne.gumypause@hcuge.ch (F.G.-P.); 2 Swiss Institute of Bioinformatics, Amphipôle, Quartier UNIL-Sorge, 1015 Lausanne, Switzerland; 3 Division of Pediatric Oncology and Hematology, Department of Women, Child and Adolescent, University Geneva Hospitals, Rue Willy-Donzé 6, 1205 Geneva, Switzerland

**Keywords:** neuroblastoma, 17q, gain, *IGF2BP1*, *NME1*, *BIRC5*

## Abstract

**Simple Summary:**

Neuroblastoma (NB) is the most frequent solid extracranial tumor in children and the most frequently diagnosed cancer during infancy. A genetic modification, a gain of 17q chromosome arm, is the most common modification in neuroblastoma. Substantial research has been performed on 17q’s role in neuroblastoma development and its clinical significance. This paper aims to make a comprehensive review of this evidence. The main findings of the review are: (1) current knowledge supports that 17q gain is involved in the development of neuroblastoma and (2) 17q gain is an important clinical marker independently and in association with other genetic modifications.

**Abstract:**

Neuroblastoma (NB) is the most frequent extracranial solid childhood tumor. Despite advances in the understanding and treatment of this disease, the prognosis in cases of high-risk NB is still poor. 17q gain has been shown to be the most frequent genomic alteration in NB. However, the significance of this remains unclear because of its high frequency and association with other genetic modifications, particularly segmental chromosomal aberrations, 1p and 11q deletions, and *MYCN* amplification, all of which are also associated with a poor clinical prognosis. This work reviewed the evidence on the clinical and biological significance of 17q gain. It strongly supports the significance of 17q gain in the development of NB and its importance as a clinically relevant marker. However, it is crucial to distinguish between whole and partial chromosome 17q gains. The most important breakpoints appear to be at 17q12 and 17q21. The former distinguishes between whole and partial chromosome 17q gain; the latter is a site of *IGF2BP1* and *NME1* genes that appear to be the main oncogenes responsible for the functional effects of 17q gain.

## 1. Introduction

Neuroblastoma (NB) is the most frequent solid extracranial tumor in children and the most frequently diagnosed cancer during infancy [1]. The median age at diagnosis is 19 months, with 90% of patients being younger than 5 years old [2]. NB can be very aggressive, accounting for approximately 15% of all pediatric cancer fatalities [3]. It can occur anywhere in the sympathetic nervous system, with the adrenal gland being the most common primary site (40%), followed by the abdominal (25%), thoracic (15%), cervical (5%), and pelvic sympathetic ganglia (5%) [3,4]. In more than 50% of patients with NB, it has already metastasized at the time of diagnosis [5].

NB is a very heterogeneous tumor in terms of location, histopathological appearance, and biological characteristics. Several factors influence the biological behavior of these tumors and are helpful in predicting disease outcomes, such as age at diagnosis, disease stage, tumor histology, and molecular and cytogenetic features such as segmental chromosomal alterations (SCAs) [4,6]. Negative prognostic markers include being over 18 months old, metastasis at diagnosis, and the presence of *MYCN* amplification (MNA), 1p loss, 11q loss, 17q gain, and DNA copy number alterations [4,7,8]. The International Neuroblastoma Risk Group stratifies patients into four prognostic subgroups based on five-year event-free survival (EFS): very low risk, with an EFS rate >85%; low risk, with an EFS rate from 75% to 85%; intermediate risk, with an EFS rate from 50% to 75%; and high risk, with an EFS rate <50% [7]. This last group has the worst prognosis, and this is despite intensive multimodal treatments, including surgery, high-dose chemotherapy with autologous bone marrow rescue, radiotherapy, and immunotherapy [7]. These patients clearly require new therapeutic strategies.

In 1984, Gilbert et al. published the first evidence of abnormalities in chromosome 17 in NB (review of 35 karyotypes of NB) [9]. A year later, Emanuel et al. identified the presence of extra 17q material in two NB cell lines by karyotyping [10]. In 1987, Christiansen et al. showed structural aberrations in chromosome 17 in a clinical case involving relapsed NB (in situ hybridization in the bone marrow cells of a girl with disseminated neuroblastoma (stage IV) at ages 9, 23, 24, and 26 months) [11]. In 1995, Caron showed that 17q gain was associated with an unfavorable prognosis in cases of NB [12]. From this preliminary evidence of 17q’s importance, the following questions remained unanswered: (1) Where and how frequently does the breakpoint occur? (2) Where is 17q translocated to? (3) Is 17q associated with other genetic features? (4) Is 17q clinically important? (5) Is 17q relevant to NB’s biology? (6) Which genes are important to these processes?

On 1 August 2023, PubMed was screened using two broad terms, neuroblastoma and 17q, which identified the 196 articles that served as a basis for this review. These articles were examined and sorted into categories to help answer the above questions. The present article reviews the clinical and biological data concerning the implications of 17q gain on the development and progression of NB.

## 2. Clinical Relevance of 17q Alteration

### 2.1. Where and How Frequently Breakpoints Occur

**Figure 1:**
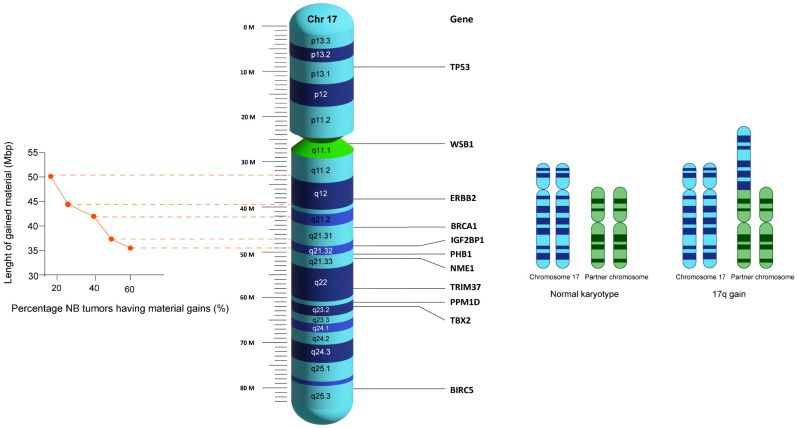
Structure, gene locations, and frequency of breakpoints in neuroblastoma tumors.

With 17q gain identified as an important region for NB development, a hunt for the culprit gene(s) was initiated. Initially, research efforts concentrated on identifying the smallest unbalanced translocation that could harbor amplified gene(s). Using a combination of fluorescence in situ hybridization (FISH) and microsatellite genotyping, the most common unbalanced translocation was found to be 17q22 to 17qter [13,14,15]. A 1998 study based on comparative genomic hybridization (CGH) by Vandesompele et al. observed partial or whole 17q gain in 81% of NB tumors (33% and 48%, respectively). Strikingly, however, partial 17q gain was found exclusively in stage III and IV tumors. The authors concluded that not all 17q gains had the same effect on NB aggressivity [16]. Later studies on primary NBs by Lastowska et al. confirmed that observation, and they identified breakpoints at 17q22 as very frequent, with 17q23.1 being the most distal breakpoint site [17,18]. Not all 17q gains had the same impact on survival, however. Patients with breakpoints distal to 17q12 (*ERBB2*) had a worse prognosis than patients with larger 17q gains. This suggests that important oncogene(s) are located in 17q23.1 or distally, and that the gain of regions proximal to 17q12 is not beneficial to tumors [17]. A similar observation was made by Chen et al. using the *WSB1* gene located at 17q11.1. Patients with higher *WBS1* expression, mapping to 17q11.1, were more likely to have a good outcome in every stage of NB. Additionally, *WBS1* expression was associated with MNA, age, and NB stage [19]. These studies were further supported by a comparison of gene expression between stage IV and stage IVS NBs. Lavarino et al. observed that stage 4S NBs expressed genes located in the 17q13 to 17q21 region, whereas stage 4 NBs expressed genes located in the 17q21 to 17q25 region [20]. Another study of 25 NB cell lines by Saito-Ohara et al. also identified 17q23 as the minimal common region of gain [21]. Further precise analysis using microsatellite genotyping and FISH on cell lines concurred with the results of the abovementioned studies, showing that breaking points were very heterogeneous. These authors concluded that gene dosage rather than gene fusion or disruption events accounted for the underlying oncogenic roles of these unbalanced translocations [22]. Using higher-resolution DNA chip technology, Mosse et al. identified the 17q24.1 to 17q24.2 region as a distal cutoff, but the majority of NB cell lines had 17q gain with 17qter [23]. Vandesompele et al. identified two important regions in 17q using array CGH (aCGH) and the gene expression profiling of 69 primary NB samples, 28 NB cell lines, and normal fetal adrenal neuroblasts. They identified 17q21.32-ter and 17q24.1-ter as hotspots for 17q gains [24]. Caren et al. used an Affymetrix^®^ single nucleotide polymorphism (SNP) chip to identify 17q23.1 as the smallest region of overlap containing the *PPM1D* gene [25]. The latest analysis of the TARGET consortium data shows an additional picture of 17q breaks. The most frequent 17q gain was from 17q21.32 to 17q25.3, which includes the *IGF2BP1* gene residing precisely on 17q21.32. 17q gain contained the q11.1, q11.2, q12, q21.1, q21.2, q21.31, and q21.32 regions at frequencies of 10%, 15%, 15%, 25%, 25%, 50%, and up to 60%, respectively [26] (Figure 1). Interestingly, 17q gain breakpoint may also depend on the presence of MNA and SCAs [27].

Chromosome 17′s structure and the locations of currently known functional genes associated with 17q gain. The graph represents the percentage of NB tumors presenting with material gain according to their respective breakpoints: the more distal parts of 17q are more frequently gained than the parts proximal to the centromere. 17q23.1-ter is the shortest region of gain.

### 2.2. Where 17q Translocates to

Early evidence suggested that 17q gain was not a random event. A study of 17 NB cell lines demonstrated that 7 of them carried a 17q translocation into 1p [28]. This was later expanded with additional cell lines, demonstrating that 12 of 29 cell lines contained a 17q translocation into 1p [29], some even involving a breakpoint at the *NF1* gene [14,30]. Analyzing 12 NB tumors, Lastowska et al. also observed frequent 17q translocations to 1p. However, other sites with 17q translocation included 9p, 10q, 11p, 14q, and 16q [31]. Translocation of 17q to 11q was later confirmed in non-MNA cell lines [32] and in two studies analyzing 27 advanced-stage NB tumors from Israel, where 17q to 11q was the most common translocation [33,34]. The study by Schleiermacher et al. on 27 NB cell lines detected a wide variety of 17q translocation sites, of which the most common was 1p. The other sites included chromosomes 2, 3, 4, 5, 6, 7, 10, 11, 12, 16, and 20 [35]. A more detailed study revealed that the breakpoint at 17q was very homogenous and located at 43.1 Mb (17q21.2 to q21.31), but the breakpoints on 11q were variable [36]. In conclusion, the most frequent sites of 17q translocations are 1p and 11q, but other chromosomes are also known recipients of 17q material. However, it remains unknown whether the site of translocation plays a role in the development of NB.

### 2.3. 17q’s Associations with Other Genetic Modifications

An early study on 58 NBs collected in Zurich showed a significant nonrandom distribution of 1p deletion and 17q gain [37]. A study by Janoueix-Lerosey et al. on 69 NBs collected in Paris concluded that 17q gain was associated with 1p36 deletion, MNA, and diploid or tetraploid chromosomal content [38]. Similarly, Souzaki et al. observed the presence of 17q gain in almost 90% (8/9) of the MNA samples but only in 40% (18/45) of the non-MNA samples [39]. Two additional studies demonstrated that 17q gain was associated with MNA [40,41]. A study by Kuzyk et al. showed that NBs with unbalanced 17q gain had high *MYCN* expression, NBs with no 17q gain showed medium *MYCN* expression, whereas whole chromosome 17q gains showed low *MYCN* expression. The first two groups were associated with a more central location of 17q within the nucleus and higher expression of 17q genes. This was further confirmed in SH-EP and GI-ME-N cells by adding *MYCN* [42]. A recent International Neuroblastoma Risk Group study demonstrated that 17q gain was associated with MNA, but an independent relationship for this association could not be established because 17q gain was also associated with 1p loss [43]. 1p deletion and MNA are not the only associations with 17q gain. In a very large study on 1091 NB tumors, of which 273 were analyzed using a single-nucleotide polymorphism (SNP) array, 17q gain occurred significantly more frequently in 11q-deleted NBs (91%) than in normal 11q non-MNA NBs (55%) [27]. Interestingly, 17q to 11q translocations appear to be associated with changes in chromosomes 3 and 7q. However, in case 17q gain was partnered with other chromosome than 11q, it was associated with 1p deletion and/or MYCN amplification [33]. Tumors of NB patients with germline *PHOX2B* or *NF1* mutations showed consistent 17q gain, with a consensus breakpoint at 17q21.31 [44]. *ALK* is another very important gene in NBs, but no associations were found between *ALK* mutations and 17q gain [45]. Interestingly, higher *HIF1α* expression was found to be associated with 17q gain, suggesting that 17q gain was involved in the metabolic reprogramming of NB [46].

### 2.4. 17q Gain’s Association with Clinically Relevant Outcomes

After the smallest region of 17q translocation had been identified, there nevertheless remained the question of whether 17q gain was clinically relevant. Very early evidence by Takeda et al. used the copy number of the *NME1* gene located on 17q21.33 to confirm the association between 17q gain and poor prognosis [47]. Two additional small-scale studies, with 18 [48] and 29 [49] NB patients, reported a high frequency of up to 90% of 17q gain and its association with poor prognosis [48,49]. Larger studies followed these initial efforts: one by Lastowska et al. [50] on 45 NBs and one by Brown et al. [51] on 104 NBs, all collected at the U.K. Children’s Cancer Study Group, and one by a German research group in Munster [52] on 53 NBs. All three studies associated 17q gains with a poor prognosis and a high relapse rate [50,51,52]. Indeed, they also found 17q gain to be associated with 1p deletion and MNA, which makes interpreting the significance of these results difficult [50,51,52]. Further independent studies from different groups based in Japan [53,54], Italy [55], France [38], Israel [34], and Sweden [56] also demonstrated associations between 17q gains and a poor prognosis, but most of those studies did not correct results for the status of MNA or 1p deletion. All this early evidence is, therefore, difficult to interpret. Because 17q gain is also associated with MNA and chromosome 1q deletion, its true contribution to clinically relevant outcomes is obscured. Some studies even described a physical association between 17q gain and the *MYCN* gene [57].

Bown et al. used univariate and multivariate analyses on a cohort of 313 NBs to demonstrate that an unbalanced 17q gain of segment 17q21-qter was associated with adverse outcomes, stage IV disease, age >1 year, MNA, and 1p deletion [58]. Next, study by Mathay showed 17q gain’s associations with metastatic disease and MNA in a cohort of 434 NB patients, 23 of which had metastasis in central nervous system [59]. Complementing these results, a study on 129 stage IV NB patients showed an association between 17q gain and adverse clinical outcomes in NBs with MNA [41]. Another large study on 202 NB patients demonstrated that a whole chromosome 17q gain was associated with better overall survival (OS) than a partial 17q gain [60]. Similarly, a very large study on 605 NB cases from Japan observed lower OS and EFS in patients with 17q gain, both across the entire cohort and in a subset of non-MNA NB patients [61]. Two further studies were performed on non-MNA NB tumors to refine information on 17q gain’s clinical significance. Caren et al. studied NBs with SCA but without MNA or 11q deletion. They showed that NBs with 17q gain had a worse prognosis than those without 17q gain [62]. Schleiermacher et al. observed the same thing but in non-MNA only NBs [63]. In a very large study on 505 non-MNA NBs, univariate statistical analysis associated 17q gain with a poorer prognosis, but no multivariate analysis was reported [64].

In contrast to the above observations, a study on 193 NBs collected in Cologne found no associations between 17q gain and outcomes. However, 17q gain was associated with stage IV disease, greater patient age, and other chromosomal aberrations (1p, 3p, 11q, and MNA). One reason why this study found no association between 17q gain and outcome could be the use of probes that were detecting for *ERBB2* and *MPO*, which are even more distal to *ERBB2*. Researchers, using these probes, may not have been able to distinguish between segmental and whole 17q gain [65]. A study by Mora et al. on 84 NB patients treated at the Memorial Sloan Kettering Cancer Center found no correlation between 17q gain and survival. As with the previous study, using microsatellite markers at 17q13 and 17q25 may not have enabled discrimination between whole and segmental 17q gain [66]. Moreover, Pezzolo et al. saw no significant impact of 17q gain on EFS in 23 Italian NB patients collected in Genova. Their study’s significance for 17q gain may be questionable, however, because of its small cohort size and focus on specific stroma-poor and resectable NBs [67]. A study in Korea also found no significance for 17q gain, but its overall incidence was far lower than normal (27% vs. >60%) [68]; therefore, both studies might have used sampling strategies that affected their statistical analyses.

### 2.5. 17q’s Clinical Relevance in the Context of Whole-Genome Analysis

The histological features of NB are less informative of outcome or prognosis than in many other cancers. Unsurprisingly, therefore, those features are also unassociated with major genetic aberrations such as MNA and the prominent chromosomal aberrations of 1p, 11q, or 17q [69]. Because these genetic modifications became a standard for clinical stratification of NB, advanced genomic methodologies proved indispensable for classifying NBs into risk groups. Lastowska et al. were among the first to attempt to classify NBs based primarily on their molecular features. One of the important genomic markers they used was 17q gain. They were able to divide NBs into three groups. Groups two and three both contained NBs with 17q gain, but they deviated significantly in prognosis depending on the presence of MNA. Interestingly, 17q gain was the only independent prognostic factor among all the factors analyzed, further highlighting its importance for NB development and clinical prognosis [70]. Vandesompele et al. published a very similar observation. Six different NB groups were observed, differentiated by their predominant genetic anomalies. Groups associated with poor prognosis in stage III or IV NBs contained either MNA, 11q, and/or 1p deletion that were significantly associated with the presence of 17q gain, again demonstrating 17q’s value in a multi-marker setup [71]. Using DNA chip technology to perform genome-wide assessments of DNA aberrations in NB, Mosse et al. used 17q status as an important marker with which to stratify NBs into four specific groups: (1) benign, (2) NBs with MNA and a 1p and 17q genomic signature, (3) aggressive NBs without MNA containing multiple structural rearrangements among which 17q gain was represented prominently, and (4) aggressive tumors with no detectable copy number variations [72]. Likewise, an improved clinical prediction strategy for NB, using a combination of *MYCN* and 17q status rather than a single marker, was also demonstrated by Suita’s group [73,74]. Spitz et al. divided 90 NBs into six groups and observed that poor prognosis was associated with SCAs, which can be identified consistently by the presence of 17q gain in addition to 2p gain or 1p, 3p, or 11q loss [75]. Schleiermacher et al. investigated the prognostic ability of numerical and segmental chromosome aberrations in 147 non-MNA NBs, including 68 cases with 17q gain. 17q gain was strongly associated with EFS and OS. Regarding the assigned CGH types, 17q gain appeared to be the best marker for classifying NB tumors in the type 2 group, which had the lowest EFS and OS. Combined with 1p deletion, Schleiermacher et al. were able to further distinguish between the type 2a and 2b groups, but no difference in EFS and OS rates was observed between these subgroups [63]. A study by Scaruffi et al. similarly demonstrated that an analysis of 17q gain could assign NBs into groups with whole or partial chromosome aberrations [76]. Finally, a large-scale study by Uryu et al. assigned 500 NBs into six distinct groups based on a detailed copy number variation analysis. 17q gain, together with 6q and 8p deletion (but not MNA and 11q deletion), were associated with a poorer prognosis in older (>5 years old) patients. In addition, 17q (together with MNA and 1p and 3p deletions) was also more frequently detected in relapsed NB [77].

In conclusion, this broad review of the scientific literature supports the idea that partial 17q gain—and not whole chromosome 17 gain—is a significant clinical event. However, 17q gain is also associated with many other genetic anomalies linked to a poor prognosis. The most prominent among these are an age of NB onset >18 months old, the presence of MNA, and SCAs. It remains unknown, therefore, whether unbalanced 17q gain has a direct or indirect impact on a patient’s prognosis. Nevertheless, studies on homogenous cohorts suggest that the impact could be direct [41,61,62,63].

### 2.6. Alternative Methods for Identifying 17q Gain

During the last 30 years of research on 17q gain in NB, multiple methods for detecting it have been developed, both for research and clinical diagnostic uses. Morowitz et al. developed a duplex polymerase chain reaction (PCR) assay for detecting unbalanced 17q gain in tumors [78]. Combaret et al. developed a PCR-based method to detect the quantity of 17q using circulating DNA in the blood. Although this method is not currently used in clinical practice, it could be useful for detecting NB in broad screening protocols because of 17q gain’s frequency [79]. Ishii et al. developed a rapid droplet digital PCR method to detect 11q in NB samples, but they suggested the same technology could be reliably used for analyzing 17q [80]. FISH is frequently used in cytological laboratories to detect chromosome amplifications, deletions, and translocations. However, multiplex ligation-dependent probe amplification (*MLPA*) was also successfully used for detecting 17q gain and other genomic aberrations in a large cohort of 202 NBs, demonstrating an association between 17q gain and unfavorable NB histology and 11q deletions [81]. CGH has provided a powerful new approach for identifying new and previously known genomic imbalances [82,83]. When comparing MLPA to aCGH, Combaret et al. demonstrated that aCGH was more reliable, less work-intensive, and cheaper [84]. However, comparing FISH and aCGH demonstrated that FISH was still more sensitive in detecting 17q gain [85]. More recently, exome sequencing was demonstrated to be more effective for detecting 17q gain than either a standard SNP array [86] or FISH [87].

## 3. The Biological Relevance of 17q Gain

### 3.1. Cellular Development of NB and 17q

When 17q gain occurs remains a controversial question. Extensive neonatal screening for NB in Japan demonstrated that patients found with NBs had an overall good prognosis. Interestingly, only 9% (3/33) of the NBs found in this study showed 17q gain, and other chromosomal aberrations were also very infrequent [88]. A study on the CLB-Bar NB cell line, obtained from primary and relapsed tumors, suggested that 17q to chromosome 4 translocations might be late events because they only occurred in relapsed tumors and after 17q to 1p translocation [89]. To further complicate the issue, results obtained by Betts et al. suggested that the appearance of 17q gain was variable [37].

On the other hand, other studies have claimed that 17q gain is an early event. This is a reasonable assumption given the very high frequency of 17q gain. A large study on 270 NBs demonstrated that 17q gain was indeed an early event, preceding all other chromosomal aberrations except probably 1p loss and the appearance of double minute chromosomes [90]. Later, using the enhanced fused lasso latent feature model (E-FLLat) to predict the path of metastatic NB development, 17q gain appeared to be one of the first events in tumor evolution [91]. These observations were supported by Krona et al., who demonstrated that 17q gain, together with *PHOX2B* mutations, might be one of the early events in NB development [92].

A substantially lower frequency (20%) of 17q gain was detected in analyses of infant NBs than in the NBs of older patients [53], for which the reported prevalence varies from 33 to 65%. Accordingly, when looking solely at NB patients over 10 years old, no MNA or 1p36 deletion were discovered, but 11q deletion (8/13) and 17q gain (7/11) were frequently detected in the high-risk group that had worse prognosis [93]. These results were supported further by Italian, Spanish, and Canadian studies on preadolescent, adolescent, and young adult NB patients that showed higher numbers of SCAs, including 17q gain and 11q deletion, but lower frequencies of MNA [94,95,96].

Morphologically, 17q gain has no impact on the histological features of NB cells [69]. Interestingly, it was observed that Schwann and neuroblastic cells come from the same precursor cell that already contains genetic modifications [97]. Likewise, ganglioneuroblastoma (GNB) is similar to NB in its genetic features, including the presence of 17q gain [98,99]. However, the ganglioneuromatous component of nodular GNB has been demonstrated to have 17q gain in 50% of cells, compared to the neuroblastoma component that showed a 100% 17q gain [100]. In late-stage tumor development, NB metastases retain their 17q gains when comparing to primary tumors [101]. The lymphatic densities of NBs were also associated with 17q gains [102]. Another important factor for tumor development is ploidy, which appears to be very important for the development of NB risk. Tomioka et al. found concordance between the disomy of chromosomes 1 and 17. NB tumors with trisomy of chromosome 1 were consistently found to have mixed populations of cells with trisomy or tetrasomy of chromosome 17. In addition, disomy of chromosome 1 was associated with segmental 17q gain [85]. In a very large study on 369 NBs with low (< 50%) NB tumor cell content, the researchers demonstrated that patients with high partial genetic instability (PGI) had poorer prognoses, and 17q gain was used as a marker for the detection of PGI [103].

### 3.2. Functional Gene Studies

Along with identifying the consensus breaking point of 17q gain, functional gene studies focused on proving the function of 17q gain by identifying the genes involved in the development and aggressiveness of NB. Early evidence for the functional involvement of 17q gain came from studies on cell lines that transited from stage IVS to stage IV, gaining 17q22 translocation but not undergoing 1p deletion or *MYCN* amplification [104]. Evidence also came from a case report about a patient with germline 17q to 1p36 translocation who developed NB [105]. Later, through combined advanced genomic and gene expression analysis, researchers demonstrated the importance of different genes on 17q gain. Lastowska et al., for example, demonstrated that genes located on 17q, and important for NB survival, were also found to be expressed on mouse orthologue of human 17q [106]. In addition to this, Lavarino et al. observed that IVS NBs expressed genes located in regions 17q13 to 17q21. In contrast, stage IV NBs expressed genes located in regions 17q21 to 17q25 [20]. A large transcriptomic study demonstrated that NBs mainly fall within global cancer signature 18, which is determined by the presence of 17q gain and *MYCN*. This signature mainly demonstrates higher expression of the mitochondrial ribosome and genes associated with electron transport [107]. Other studies that demonstrated the importance of 17q genes in the development of NB include two studies by Ramani et al. that suggested that 17q gain might be associated with cell proliferation index [108,109]. The study by Schleiermacher et al. reported that 17q gain, along with 1p deletion and *MYCN* amplification, resulted in an exchange of genetic material that was replicated early in the cell cycle’s S-phase [35]. Wong et al. demonstrated dose-dependent relationships between the copy number gains of *PI3K* and *STAT* family genes on 17q and the susceptibility of NB to cell cycle and PI3K inhibitors [110].

17q gain’s importance in NB development was also demonstrated using animal models. Numerous studies demonstrated gains in the mouse 11q chromosome, which is homologous to human 17q in the NB mouse model [111,112,113]. Studies concluded that genes at the breakpoint must be important for NB development [112], thus supporting the evidence from human samples that MNA was associated with 17q gain [114]. Similar results were obtained using *ALK*-driven NB mouse models: NBs in this model also developed 17q gain and MNA [115]. Interestingly, in the same model, the co-expression of *ALK* and *MYCN* produced minimal chromosome aberrations, suggesting less need for additional mutations and explaining why no association was observed between *ALK* and 17q gain in human NB samples [45]. A new, chemo-resistant mouse model based on *MYCN* overexpression and using additional cyclophosphamide chemotherapy that produced metastases demonstrated that 17q gain was instrumental in the transformation process [116]. De Wyn et al. used the TH-MYCN NB mouse model to identify the *CBX2*, *GJC1*, and *LIMD2* genes involved in the early stages of NB development (34638267). In addition to these models, researchers demonstrated that NB xenografts retained 17q gain after transplantation into mice [117,118] or as patient-derived organoids [119]. They also showed that tumor-initiating cells grown in a serum-free medium were able to establish in vivo tumors that retained 17q gain [120].

In conclusion, the evidence obtained from cellular and functional gene studies supports the hypothesis that 17q gain has an important functional role in NB development.

#### 3.2.1. BIRC5 (Survivin)

**Figure 2:**
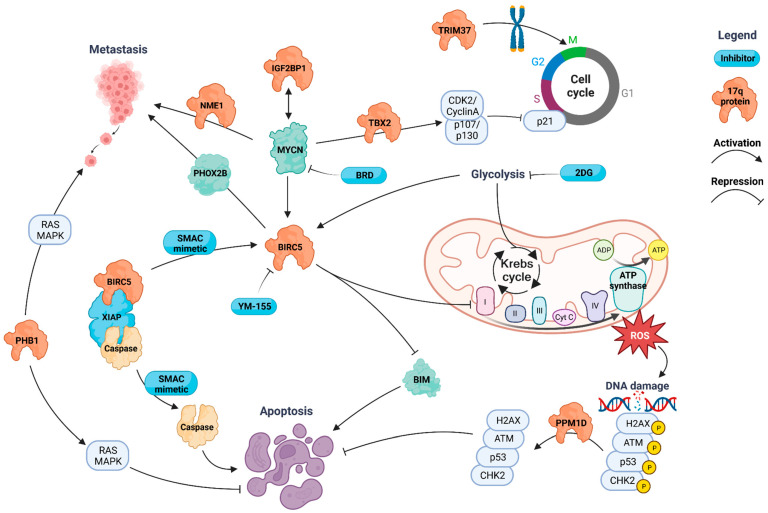
The functions of the genes located on the 17q gain.

Early functional studies focused on *BIRC5* (17q25.3), which was an obvious candidate due to prior knowledge of its involvement in apoptotic cell death (Figure 2). It was demonstrated that high *BIRC5* expression was associated with poor prognostic factors and that it promoted cell survival in NB [121]. The inhibition of BIRC5 was demonstrated to be a promising new treatment for NB [122,123]. Later, it was demonstrated that this association was independent of 17q gain. Silencing *BIRC5* leads to apoptosis through the multinucleation of NB cells and the mitotic catastrophe that activates p53 and apoptosis via CASP2 [124] (21859926). Additionally, BIRC5 represses BCL2L11 (Bim) and inhibits oxidative stress due to respiration by inhibiting respiratory complex-1. Loss of energy through respiration is replaced by increased glycolysis, which is a convenient target for neutralizing BIRC5′s effects [125]. An inhibitor of glycolysis, 2-deoxy-d-glucose (2-DG), inhibits BIRC5 via its degradation through E3-ubiquitin ligase Parkin, a target of PINK1. BIRC5 degradation activates Beclin-1, which activates autophagy and increases cell death. The knockdown of *Packin*, on the other hand, reduced the sensitivity of NBs expressing *BIRC5* to glycolysis inhibition [126]. 2-DG also synergizes with the SMAC mimetics that induce apoptosis by releasing caspases from XIAP complexes. However, XIAP complexes also sequester BIRC5, and the SMAC mimetics-mediated release of BIRC5 can lead to the fragmentation of mitochondria, decreased oxidative phosphorylation, and thus prevention of reactive oxygen species, and increased glycolysis, all of which promote cell survival. This unwanted effect of SMAC-mimetics treatment could be prevented by 2-DG [127].

#### 3.2.2. NME1 (NM23-H1)

Another gene investigated early on was *NME1*, historically known as *NM23-H1*, which is also located at 17q21.33. Initial investigations demonstrated higher *NME1* and *NM23-H2* expression in MNA cells (Figure 2). Both genes were induced 4 h after *MYCN* was switched on [128]. Vandesompele et al. further demonstrated the prognostic power of *NME1* and *NM23-H2* expression levels, with higher expression being associated with a poorer prognosis [24]. In addition, the splicing that leads to the expression of different gene variants might be important in regulating the activity of NME1 and NM23-H2 because NM23-LV, which is involved in this mechanism, was found to be highly expressed in tumors [129]. Further functional testing of the *NME1* gene demonstrated an association between *NME1* expression and NB cell migration and differentiation [130]. The suppression of cdc42, which blocks cellular differentiation, was identified as a crucial target of both MYCN and NME1 or NM23-H2. However, a low level of cdc42 activity is needed nonetheless because silencing cdc42 activates massive apoptosis [131]. Importantly, cdc42 appears to be regulated by Rho guanosine triphosphates (GTPases), which are central signaling pathways during the development of the nervous system and in neuritogenesis, together with cdc42 [41].

#### 3.2.3. IGF2BP1

*IGF2BP1* is located close to *NME1* at 17q21.32. Very recently, two large functional studies investigated *IGF2BP1* function in NB, expanding on previous work by Bell et al. that demonstrated the association between *IGF2BP1* and *MYCN* expression and a statistically significant lower OS among patients with high *IGF2BP1* expression [132] (Figure 2). Hagemann et al. demonstrated a feed-forward interaction between IGF2BP1 and MYCN through the promotion of 17q/2p chromosomal gains. Conditional *IGF2BP1* expression in sympathoadrenal tissue of mouse model resulted in a 100% incidence of NB and shorter disease latency when IGF2BP1 and MYCN were co-expressed. Developed NBs showed 2p/17q gains and high *MYCN*, *BIRC5*, and Phox2b expression. Interestingly, simultaneous targeting of IGF2BP1 by BTYNB and MYCN, or BIRC5 by BRD or YM-155, respectively, showed beneficial effects in vitro and in vivo [133]. In the same year, Dhamdhere et al. used an in vivo mouse model to demonstrate that *IGF2BP1* was also involved in the promotion of NB metastases. Metastases are promoted through small extracellular vesicles [134], which were recently demonstrated to have an important role in the metastasis process through their regulation of cancer’s microenvironment, the modulation of immune response, and the transmission of drug resistance and oncogenic properties to surrounding cells [135,136].

#### 3.2.4. PPM1D

A screening exercise performed on 25 NB cell lines identified minimal chromosomal region of gain at 17q23. Next, seven genes in that region, including *PPM1D*, were identified as consistently upregulated in these 25 NB cell lines. However, only PPM1D higher expression was significantly corelated with poor clinical prognosis in the cohort of 32 NB patients [21]. Silencing *PPM1D* resulted in the suppression of cell growth as it also activated apoptosis [21] (Figure 2). These results were further reinforced by demonstrating that the PPM1D inhibitor, GSK2830371, was more effective against wild-type *TP53* than against mutated *TP53* [137]. PPM1D is a phosphatase that dephosphorylates p53, CHK2, H2AX, and ATM, and it therefore negatively regulates the cell cycle. Hypothesizing that mutations in *TP53* might destabilize the genome and be a causal factor for 17q translocations, Vogan et al. searched for mutations in *TP53* and 17q translocations [138]. Finding very few *TP53* mutations, researchers concluded that the inactivation of *TP53* was not a necessary factor for the occurrence of 17q translocations [138]. However, wild-type *TP53* could serve as a target for reactivation through the inhibition of PPM1D, as Richter et al. demonstrated. In addition, the PPM1D inhibitor, GSK2830371, synergized well with doxorubicin and carboplatin by inducing apoptosis through Caspase 3/7 [137]. These results were further reinforced in a study on another PPM1D inhibitor, SL-176, which demonstrated similar efficacy to known p53 reactivators such as Nutlin-3 and RITA [139,140].

#### 3.2.5. TBX2

*TBX2*, located on 17q23.2, was identified as a super-enhancer marked transcription factor in NB, and it is a constituent of NB’s core regulatory circuitry (CRC). *TBX2*, when combined with *MYCN*, was shown to activate FOXM1, which in turn activates CDK2/cyclin A and p107/p130, resulting in the inhibition of the p21-DREAM complex (Figure 2). On the other hand, the combined *MYCN*/*TBX2* knockdown forced cell growth arrest. The transcriptional addiction to TBX2/MYCN was also targeted using combined CDK7 and BET bromodomain inhibition that showed synergistic repression of cell viability, CRC gene expression, and activation of the p53 pathway response [141,142].

#### 3.2.6. PHB1 (Prohibitin 1)

RAS-MAPK activation is one of the main pathways contributing to the development of NB [143]. PHB1 facilitates the activation of c-RAF by RAS [144] (Figure 2). Using the whole-genome and RNA sequencing of NBs, MacArthur et al. demonstrated that high *PHB1* (17q21.33) expression correlated with a poor prognosis and that a loss of gene expression programs promoted neuronal development and differentiation. In addition to that, PHB1 depletion reduced ERK1/2 phosphorylation, induced differentiation and apoptosis, and slowed cell cycle progression. On the other hand, ectopic *PHB1* expression increased cell proliferation and reduced markers of differentiation and favorable outcomes [145].

#### 3.2.7. TRIM37

TRIM37 is a PLK4 ubiquitination protein that is important for the proper assembly of centromeres. The gene is located on 17q22 and has been described as upregulated in many different cancers, particularly NB. Inactivating *TRIM37* improved acentrosomal mitosis because its absence allowed for the self-assembly of microtubule-organizing centromere-independent PLK4 condensates [146] (Figure 2). On the other hand, ectopic *TRIM37* expression inhibited acentrosomal spindle assembly by degrading its essential component, CEP192. Interestingly, the antitumoral activity of centrinone, a selective PLK4 inhibitor, is dependent on TRIM37. It was demonstrated that higher levels of *TRIM37* expression render NB cell lines sensitive to centrinone by triggering selective mitotic failure [146].

#### 3.2.8. Noncoding RNAs

The *ncRAN* gene is a noncoding RNA gene located at 17q25.1. CGH and expression profiling in 70 NB patients showed that high *ncRAN* expression was associated with a poor prognosis, even after multivariate analysis. Ectopic *ncRAN* expression induced the transformation of NIH3T3 cells in soft agar. In contrast, silencing *ncRAN* in SK-SY5Y significantly inhibited cell growth [147]. Another long noncoding RNA, 6p22lncRNA, was demonstrated to regulate the stability of CHD7, which modulates the USP36 localization that is responsible for *Sox9* expression. Both *SOX9* and *USP36* are found on 17q, demonstrating the apparent interplay between chromosomes 6p22 and 17q [148].

#### 3.2.9. BRCA1

*BRCA1* is located on 17q21.31 and is frequently mutated in NB [149]. A comprehensive study by Varkhedi et al. demonstrated an association between reduced *BRCA1* copy numbers and reduced OS [150]. Functionally, *BRCA1* was demonstrated to be involved in the MYCN-dependent transcription termination of cell cycle-regulated genes in the absence of a nuclear RNA exosome. The absence of an RNA exosome leads to stalled replication forks, double-strand breaks, and S-phase cell cycle arrest [151,152]. BRCA1 and BARD1 are also responsible for the ubiquitination of p50. The loss of p50 ubiquitination is responsible for the S-phase progression and chromosome breakage that can drive tumorigenesis [153]. Interestingly, SNPs in the *BARD1* promoter region, which are associated with lower expression of BARD1, are also associated with stage III/IV NB and NB of adrenal origin [154,155]. BRCA1 is also implicated in the BRCA1/Chk1/p53 pathway-mediated upregulation of p21 (Cip1/Waf1) [156]. The very low frequency of point mutations in *TP53* further highlights the importance of BRCA1 dysregulation in the loss of this checkpoint mechanism [47,157]. *TP53* resides on the 17p arm, which is not frequently lost in NB and is independent of 17q gain [47]. How exactly *BRCA1* expression is downregulated in NB is not fully understood, but it is known that genes at translocation breakpoints can undergo downregulation due to the inaccurate repair of such events [158,159].

#### 3.2.10. ERBB2

*EBRR2* (*Her2*) is located on 17q12 and is most notably associated with breast, ovarian, endometrial, and uterine tumor development [160]. This gene is a member of the tyrosine kinase family but lacks the typical growth factor binding domain. The ERBB2 protein interacts with other tyrosine kinases to help transmit signals through the mitogen-activated protein kinase and along the PI3K signaling cascade [161]. Although frequently mutated in other cancers, surprisingly low frequencies of mutations in *ERBB2* have been detected in NB [157]. Furthermore, higher ERBB2 expression because of the amplification of the gene does not appear to be particularly important for NB development since no association between high ERBB2 expression and clinical outcomes has been observed [162]. On the contrary, some studies suggest that this amplification might be detrimental, although the amplification of ERBB2 might simply be an indication of a more favorable whole-chromosome 17q gain rather than a direct negative effect of *ERBB2* amplification. A study by Izycka-Swieszewska et al. demonstrated that tumors with higher *ERBB2* expression had a better prognosis than tumors with low or no expression [163], while a study by Wilzen et al. showed that high expression of ERBB3 was associated with a high expression of ERBB2, and that the expression profile of NB was more related to nonaggressive GNB and ganglioneuroma [164]. The *ERBB2* I655V mutation was recently described in approximately 40% of refractory NB after standard multimodal therapy, suggesting that it might be an important late-developmental adaptation to chemotherapy playing a role in chemoresistance [165]. This result was also supported by in vitro experiments on IMR32 cells which showed that increased ERBB2 expression after cisplatin treatment helped those cells grafted more frequently to chicken chorioallantoic membrane [166].

## 4. Conclusions

In conclusion, the evidence presented in this work robustly supports the functional importance of 17q gain in the development of NB but also as a clinically significant marker. Partial 17q gain has been shown to be associated with other clinically important factors, particularly SCA, 1p and 11q deletions, and MNA. Nevertheless, many studies also showed 17q gain as an independent prognosis factor. However, it is important to distinguish the difference between whole and partial chromosome 17q gains. The most important breakpoints appear to be at 17q12 and 17q21. The breakpoints proximal or distal to 17q12 appear to distinguish between whole and partial chromosome 17q gain, respectively, whereas 17q21 is a site of *IGF2BP1* and *NME1*. Although our methodology provided us a better understanding of 17q gain’s clinical and biological relevance in NB, numerous issues still require clarification. Functional studies suggest that overexpression of distal genes such as *IGF2BP1*, *NME1*, and *BIRC5*, through the gain of genetic material, are responsible for the functional effects of 17q gain. Nevertheless, the importance of other genes distal to 17q21 cannot be excluded. For now, 17q gain’s precise contribution to the development of NB is still not completely elucidated. Most studies appear to agree that 17q gain occurs after 1p36 deletion, but it is not clear whether it contributes to the establishment of MNA. The results support the view that 17q gain helps drive MYCN expression through IGF2BP1, but it could also help cells overcome the oncogenic stress associated with a gradual increase in MYCN expression. The other key question that needs to be resolved is the connection between 17q gain and SCA. It is currently unclear whether 17q gain is a product of SCA or contributes to this phenomenon, given that genes like *BRCA1*, *TRIM37*, and *PPM1D* could contribute to chromosomal instability. It also remains unclear whether larger 17q deletions are favorable because they represent non-SCA phenotypes or whether the loss of certain genetic material is detrimental to cancer cells.

## Data Availability

Not applicable.
